# Physical activity from the perspective of older adults: a convergent mixed-method study

**DOI:** 10.1186/s12877-024-05362-x

**Published:** 2024-09-18

**Authors:** Anna Nilstomt, Johanna Gustavsson, Linda Beckman, Charlotte Bäccman, Finn Nilson, Stefan Wagnsson, Erik Wästlund

**Affiliations:** 1 Service Research Center (CTF), Karlstad University, Karlstad, Sweden; 2 Department of Political, Historical, Religious and Cultural Studies, Karlstad University, Karlstad, Sweden; 3 Center for Societal Risk Research, Karlstad University, Karlstad, Sweden; 4 Department of Public Health, Karlstad University, Karlstad, Sweden; 5 Department of Health Services Research, Management & Policy, University of Florida, Gainesville, FL USA; 6 Department of Educational Studies, Karlstad University, Karlstad, Sweden

**Keywords:** Active aging, Physical activity, Community-dwelling older adults, COM-B model, Convergent mixed-methods, SDG 3: Good health and well-being

## Abstract

**Background:**

Older adults are insufficiently physically active, despite its importance for healthy aging. To develop appropriate physical activity interventions, it is necessary to understand their physical activity. This study applies a theoretical perspective, the COM-B model, and a mixed-method design to examine what influences older adults’ physical activity levels with three questions: (1) What individual and external factors predict older adults’ physical activity levels? (2) What do older adults perceive as influencing their levels of physical activity? (3) To what extent do the quantitative results on older adults’ physical activity levels agree and disagree with the qualitative findings on older adults’ physical activity levels?

**Methods:**

A convergent mixed-method design was used with questionnaire (*n* = 334) and interview (*n* = 14) data from adults 65 years and older. Regression analyses were used for quantitative measurements: physical activity, age, subjective socioeconomic status, health status, capability, opportunity, motivation, and depression. Content analysis was applied to the qualitative data. The two forms of data were then integrated to provide greater insights than would be obtained by either dataset separately.

**Results:**

The regression analyses showed that previous physical activity, current motivation, health status, and age significantly predicted older adults’ physical activity levels. The content analysis revealed that participants addressed all subcomponents of the COM-B model, indicating its pertinence in understanding how older adults discuss their current physical activity levels. The integrated findings showed convergent and divergent results. Overall results indicated that previous physical activity engagement, present motivation, capability, and opportunity influenced older adults’ physical activity levels.

**Conclusions:**

This study is the first to use this mixed-methods design to examine factors influencing physical activity levels among older adults living in rental apartments with community hosts. The integrated result reveals convergence for findings on motivation and physical capability but divergence on psychological capability, opportunity, and previous physical activity. The findings underscore a complex interplay of factors influencing older adults’ physical activity levels and indicate relevance for the COM-B model. The results can guide future research on theoretically informed interventions to promote physical activity and healthy aging. Future research should clarify the role of opportunity for older adults’ physical activity.

Physical activity (PA) is vital for healthy aging as it promotes mobility and independence [1] and reduces declines in health and functioning [2]. The World Health Organization (3, p. vii) defines PA as “any bodily movement produced by skeletal muscles that requires energy expenditure”, which implies various activities, such as leisure-time activities like walking and gardening, transportation by bike, household chores, occupational tasks if the person still works, or planned exercise.

Adults aged 65 years or over are recommended to engage in at least 150 min of aerobic activities per week, perform muscle-strengthening exercises twice a week, and practice their balance three days a week [3], or at a minimum be as active as their abilities and conditions allow [3, 4]. Previous research has shown that older adults have a more flexible attitude towards PA, ranging from any activity moving the body and mind to strictly planned activities outside the home [5]. In Sweden, fewer than 60 percent of older adults aged 65–84 meet the aerobic recommendations [6], meaning many older adults are insufficiently active. Also, the oldest adults are less likely to engage in PA than those of younger ages [7], which raises the question of what influences older adults’ PA and how it can be promoted in society? The answer to these questions could improve healthy aging and support the Agenda 2030 sustainable development goal of good health and well-being [8].

Previous research has identified several individual and external factors that influence older adults´ levels of PA, for example, being physically active earlier in life [9], wanting to continue with PA [10], and perceiving their health status as good [11, 12]. Also, participating in group activities [5], having a supportive social network [13], access to facilities, and favorable weather conditions seem to promote older adults’ level of PA [13]. Factors that seem to decrease the likelihood of PA engagement among older adults are, for example, physical deterioration of the body [14], pain, fatigue, fear of falling [15], low self-confidence [14], low subjective socioeconomic status (SES) [16, 17] and depressive symptoms [18]. However, to gain a deeper understanding of older adults’ PA engagement, applying a theoretical perspective, such as the COM-B model, is valuable [19] and increases the chances of designing effective interventions [20]. Thus, the different individual and external factors linked to older adults’ PA levels can be understood through the COM-B model [19].

The COM-B model refers to three components – capability, opportunity, and motivation – that must be present to generate a specific behavior, such as PA [19, 21]. Capability and motivation relate to intrinsic factors, whilst opportunity relates to external factors. More specifically, capability concerns the individual’s physical and psychological capacity to engage in a behavior, for example, skills, knowledge, and thought processes; opportunity can be social or physical possibilities that allow a behavior to occur; and motivation is a mental process that can be either reflective or emotional and that energizes the targeted behavior as well as including goal-setting, decision-making, habits, and emotional responses [21]. Each component can directly impact behavior, and opportunity and capability can also indirectly impact behavior through motivation. The behavior can also impact capability, opportunity, and motivation [21].

The COM-B model is commonly related to PA [22–24] but has only to a limited degree been used for understanding older adults’ levels of PA. A review by Meredith et al. [25], which included qualitative studies on older adults, mapped the results to the COM-B model to better understand what influences older adults’ PA engagement. This secondary analysis revealed that all the COM-B components interacted and affected older adults’ PA engagement and that opportunity was the most frequently identified component [25]. Although reviews can be valuable for summarizing research results, they rely solely on secondary data instead of primary data, which may limit understanding nuances and the ability to establish relationships in the data [23]. Therefore, it is important to conduct more studies on the levels of PA in older adults, using the COM-B model. These studies should preferably use a mixed-methods approach since the convergence of two forms of data brings greater insights than would be obtained by either type of data separately [26]. Qualitative methods can provide deeper insights and a more nuanced view of a phenomenon, while quantitative methods enable statistical generalizability [27].

By understanding what influences older adults’ PA in relation to the COM-B model and two datasets, we can better understand the phenomenon of PA in this population. The COM-B model is useful for analyzing behavior as it is part of the theoretical framework for behavior change interventions [19]. Thus, we can acquire valuable knowledge that can be used to create and improve interventions that support older adults’ PA engagement. Ultimately, this can contribute to healthier aging.

## Methods

This study aimed to examine what influences older adults’ levels of PA and how it could be understood in terms of the COM-B model and by means of a convergent mixed-method design. It is a design where quantitative and qualitative data are collected approximately simultaneously and analyzed separately before integration [26]. In this study, the two datasets were equally emphasized [26]. This study used quantitative observational data from a cross-sectional study to understand older adults’ PA levels through the COM-B model. The qualitative data from interviews explored PA among older adults. The reason for collecting both quantitative and qualitative data was to converge the two forms of data to bring greater insights into the research problem than would be obtained by either type of data separately. Qualitative methods can provide deeper insights and a more nuanced view of a phenomenon, while quantitative methods enable statistical generalizability to a greater degree [27]. A pragmatic approach was applied, as this allows using both quantitative and qualitative research methods to collect the data needed to address the study’s research questions [26]. The National Research Ethics Committee approved the study before recruitment.

The aim was operationalized into three research questions:

1. What individual and external factors predict older adults’ PA levels?
2. What do older adults perceive as influencing their levels of PA?
3. To what extent do the quantitative results on older adults’ PA levels agree and disagree with the qualitative findings on older adults’ PA levels?

### Study setting

The recruited participants were 65 years or older and lived in rental apartments owned by the municipality. The apartments are specifically designed to provide independent living facilities for older adults and include a so-called ‘Trygghetsvärd’ (community host) who functions as a social support. Aside from arranging social activities like café meetings and walking groups, the community hosts clean the common areas and assist with simple tasks in the residents’ homes, such as changing light bulbs. The fact that the participants lived in rented apartments means that they differ from the Swedish average. In Sweden, owning a house is the most common housing arrangement, followed by renting an apartment and then owning an apartment [28]. The difference between the study group and the general population should be considered when interpreting the results.

### The quantitative method

A cross-sectional study addressed the first research question: What individual and external factors predict older adults’ PA levels?

#### Participants

The sample consisted of 334 community-dwelling older adults (71.3% women) in a middle-sized town in Sweden. The majority of participants (65.2%) lived alone. Age was measured as a five-year categorical variable ranging from 65–70 years to 95–100 years. The mode age category was 76–80 years. Almost half of all participants (49.4%) could be classified as sufficiently active, 17.4 percent as moderately active, and 33.3 percent as insufficiently active, according to the Godin-Shepard leisure-time exercise questionnaire [29]. The participants reported a low degree of depressive symptoms (*M* = 3.98, *SD* = 3.84, Range = 0–19). However, 24.4 percent of the sample reported six or more symptoms of depression, which, according to the cut-off score of the Geriatric Depression Scale [30], might indicate depression.

#### Procedure and response rate

A pilot survey was tested among a sample of self-recruited older adults (*n* = 6) from the local residential areas. This resulted in a shortened survey that was distributed to a total of 700 older adults in February 2022. Participants returned the survey either by pre-paid postage or through a sealed envelope that the community hosts mailed. One reminder was sent to their postbox and notes were posted on one occasion at the apartment complexes’ entrance to boost the response rate. Upon request from the older adults, the community hosts were available to assist with the questionnaires. This approach aimed to minimize dropouts caused, for example, by visual impairments. The community hosts assisted a total of three older adults. In total, 334 older adults provided informed consent to participate, resulting in a response rate of 48 percent.

#### Instruments

The Godin-Shepard leisure-time exercise questionnaire (GSLTEQ), adjusted by Godin [29], assessed the individual’s weekly PA. The participants self-reported on three items their frequencies of at least 15 min of mild, moderate, and strenuous PA per week on an eight-point scale ranging from *0* to *7 days*. The standard procedure described by Godin [29] was used to calculate the total PA score and classify individuals as either sufficiently, moderately, or insufficiently active. The total PA scale index ranges from 0–119, where a higher score indicates an increased PA level. The Cronbach’s alpha was 0.65, and the inter-item correlation was 0.39 in this study.

An adjusted version of the COM-B instrument, constructed by Bäccman, Bergkvist, and Wästlund [31], was used to assess the COM-B model [21]. It contained 12 items designed to measure capability (four items, such as “I know why it is important to be physically active”), opportunity (four items; for example, “I have access to facilities and equipment required to be physically active”), and motivation (four items, such as “I really want to be physically active”). Each item was answered on a five-point scale ranging from *strongly disagree* to *strongly agree.* The mean for each index, capability, opportunity, and motivation was used for the analysis. In this study, Cronbach’s alphas for the capability, opportunity, and motivation index were 0.80, 0.78, and 0.92, respectively.

Health-related quality of life was assessed by EQ-5D-3L [32]. The participants classified their health on five dimensions (mobility, self-care, usual activities, pain/discomfort, anxiety/depression) and three severity levels (*no, moderate, or severe problems*). The health levels were transformed into an index value by applying the Swedish value set by Burström et al. [33]. The index values ranged between 0.3402 and 0.9694, where the former represents the lowest self-rated health-related quality of life and the latter the highest. In this study, the Cronbach’s alpha was 0.60, with an inter-item correlation of 0.24.

Symptoms of depression were assessed with the Swedish version of the Geriatric Depression Scale (GDS) developed by Gottfries, Noltorp, and Nørgaard [30]. The scale is a screening instrument consisting of 20 items to be answered with a *yes* or *no*. Five items were reversed before summing a total score that ranged from 0–20, with a higher score indicating more symptoms of depression. A score of six or more indicates that depression might be suspected, whereas a score of five or lower suggests that depression is unlikely [30]. The Cronbach’s alpha was 0.86 in this study.

The individual’s perception of their subjective socioeconomic status (subjective SES) was assessed with a single item, asking the participants to rank their socioeconomic status compared to other older adults in the society on a 10-point scale ranging from *lowest* to *highest* [34].

Previous PA experiences were assessed with a single item, asking the person to “Rate the average degree to which you have been physically active (exercised/worked out) in your life up until today.” The answers were given on a five-point scale ranging from a *very high degree of physical activity* to a *very low degree of physical activity*.

The questionnaire also included demographic information (age, gender, and cohabitation). Age was measured as a categorical variable with an interval of five years (e.g., 65–70). The lowest age category was *65–70 years* and the highest was *106 years and older*.

#### Data analysis

The data were analyzed using descriptive statistics and multiple regression. The data did not indicate any outliers. Only two cases had an age of 96 years or above. Therefore, the two age categories 91–95 and 96–100 were merged into an age category of 91–100.

A hierarchical regression analysis was applied using bootstrapping to obviate skewed data. First, a hierarchical regression with three steps was completed, using forced entry within each step. The reason for this procedure was to test the COM-B model initially before adding characteristics. In the first step, opportunity and capability were included, as these variables are theorized to generate behavior and contribute to motivation. In the second step, motivation was added. The additional individual factors (previous PA, EQ-5D-3L, subjective SES, age, and GDS) were included in the third step. After that, a trimmed regression model with forced entry was completed, containing only the significant variables retained from the hierarchical regression. For both regression models, GSLTEQ was the dependent variable. The significance level was kept at an alpha level of 0.05 and cases were excluded listwise. Statistical analysis was completed with SPSS version 28.

### The qualitative method

Semi-structured interviews addressed the second research question: What do older adults perceive as influencing their PA levels?

#### Participants

The sample (*N* = 14) is a subset of the quantitative sample. Among the participants in the qualitative sample, seven were women, 12 lived alone, and nine reported age 80 or younger. The mode age category was 86–90 years. Among the participants, four were classified as sufficiently active, four were deemed moderately active, and six were classified as insufficiently active.

#### Researcher description

The first and second authors conducted the interviews (*n* = 10 and *n* = 4, respectively). The initial coding was done by the first, second, and third authors (*n* = 8, *n* = 2, *n* = 4, respectively). The coding was calibrated through discussions among the coders, and when uncertainty remained, all the authors were consulted. After that, the first author iteratively refined the coding for all 14 interviews. Our preunderstanding was managed by self-reflection through notes. The research team has experience in quantitative and qualitative studies regarding older adults and behavior change, and its members are from psychology, nursing, public health, sports science, and physical therapy.

#### Procedure and data collection

The older adults who had participated in the quantitative data collection and had agreed to be contacted for future research were approached. The participants were selected based on their gender (male, female), age (65 to 80 years, 80 years and older), and level of PA (insufficient, moderate, sufficient) derived from their survey answers for sample diversity. A four-cycle purposive sequential sampling procedure was used to contact 35 potential participants, 14 of whom consented in writing to be interviewed. Hence, the included sample is based on the maximum number of consented participants. There was no researcher-participant interaction before the data collection.

One-on-one semi-structured interviews were conducted in March and April 2023. The interviews took place either in the participant’s home or in a secluded area at the community hosts’ office, without the presence of non-participants. The participants chose the option that was most comfortable for them. The participants were informed about the purpose of the study, their right to refrain from answering questions, and that their participation was voluntary.

An interview guide with open-ended questions, organized into four themes, was developed, and follow-up questions were tailored during each interview based on the participant’s response. The themes included the participants’ definition of PA, their experience with PA today and previously, and their thoughts on maintaining PA. While the core of the interview guide remained consistent, the questions were nuanced and refined throughout the interview process in response to participants’ answers. The interviews were audio-recorded and transcribed verbatim. The average duration for the interviews was 70 min. The range of interview length was 31–105 min.

To ensure trustworthiness, we spent lengthy time with the specific population, made notes throughout the research process, and applied investigators’ triangulation by being multiple interviewers and coders discussing the results within the research team.

#### Data analysis

Entire transcripts were analyzed using qualitative content analysis, a method for systematically interpreting text content through coding and identifying patterns [35]. The method described by Graneheim and Lundman [36] was applied to the transcripts with the adjustment of not condensing the meaning units. The text was initially read as a whole, then coded and sorted into categories using abductive reasoning. Through this process, the COM-B model was identified as a relevant framework for categorizing the codes based on the entire sample’s quotes. The analysis was completed in NVivo 14.

### The mixed-method

The quantitative results were merged with the qualitative findings to address the third research question: To what extent do the quantitative results on older adults’ PA levels agree and disagree with the qualitative findings on older adults’ PA levels?

#### Data analysis

Before integrating the two datasets through methodological triangulation, each part was independently completed according to its methodological quality standards to ensure trustworthiness [26]. After identifying quantitative and qualitative findings, the research team compared the results, discussing convergence and divergence in content by reviewing constructs, scale items, and verbal statements. We also reflected upon discrepancies in findings. Those reflections can be located in the discussion section of this paper. A joint table was created to array the results. The table includes only the statistically significant predictors and the main categories identified in each dataset.

## The quantitative results

To investigate the first research question – What individual or external factors predict older adults’ PA levels? – a hierarchical regression model was used, with capability, opportunity, motivation, previous PA, EQ-5D-3L, subjective SES, age, and GDS as predictors, and GSLTEQ as the dependent variable. Descriptive statistics (sample size, mean, standard deviation, median, and range) of the variables are shown in Table 1.

**Table 1:** Descriptive information about the variables in the regression analysis

Variable	n	M	SD	Mdn	Min-Max^a^
GSLTEQ	324	34.96	27.52		0.00–119.00
Capability	319	3.94	.96		1.00–5.00
Opportunity	314	3.06	1.16		1.00–5.00
Motivation	321	3.86	1.09		1.00–5.00
Previous physical activity	321			3^b^	1.00–5.00
EQ-5D-3L	317	.87	.10		.43-.97
Subjective SES	318	6.07	1.92		1.00–10.00
Age	326			3^c^	1.00–6.00
GDS	326	3.98	3.84		0.00–19.00

*N* = 334. *GSLTEQ* The Godin-Shepard leisure-time exercise questionnaire, *EQ-5D-3L* health status, *Subjective SES* Subjective socioeconomic status, *GDS* The Geriatric Depression Scale

^a^Min–Max = based on observed values

^b^3 = In-between low and high degree of previous physical activity

^c^3 = 76–80 years

The hierarchical regression model was significant, *R*^*2*^ = 0.38, *F* [8, 294] = 24.52, *p* < 0.001, although only the variables motivation, EQ-5D-3L, age, and previous PA were significant (see Table 2). The participants’ previous PA had a higher semipartial correlation value (*sr* = 0.30, *p* < 0.001) than EQ-5D-3L (*sr* = 0.17, *p* < 0.001), motivation (*sr* = 0.12, *p* = 0.008) and age (*sr* = -0.12,* p* = 0.01).

**Table 2:** Hierarchical regression result for physical activity

Variables	B	95 % CI for B		SE B	β	R^2^	Δ R^2^
		LL	UL				
Step 1						.18	.18***
Constant	-13.43*	-24.32	-3.24	5.41			
Capability	10.51***	7.20	14.12	1.76	.36		
Opportunity	2.31	-1.13	5.56	1.63	.10		
Step 2						.24	.06***
Constant	-23.51***	-34.98	-12.37	5.57			
Capability	6.54**	3.03	10.05	1.75	.22		
Opportunity	.41	-2.70	3.59	1.64	.02		
Motivation	8.14***	5.34	11.16	1.53	.31		
Step 3						.40	.16***
Constant	-74.12***	-103.75	-38.81	16.63			
Capability	1.71	-2.27	5.26	1.89	.06		
Opportunity	1.66	-1.54	4.95	1.60	.07		
Motivation	4.27**	1.47	7.18	1.41	.16		
Previous physical activity	10.86***	7.26	14.27	1.80	.35		
EQ-5D-3L	66.27***	29.17	98.47	17.29	.25		
Subjective SES	-.76	-2.50	.75	.83	-.05		
Age	-2.48*	-4.32	-.89	.89	-.12		
GDS	.63	-.20	1.40	.42	.09		

*N = *303. *GSLTEQ *The Godin-Shepard leisure-time exercise questionnaire, *EQ-5D-3L *health status, *Subjective SES *Subjective socioeconomic status, *GDS *The Geriatric Depression Scale. Confidence interval and standard error are based on 1000 bootstrap samples*. CI *confidence interval, *LL *lower limit, *UL *upper limit

* p <.05. ** p < .01. *** p < .001

Therefore, a trimmed regression model that only included the significant variables was completed (see Table 3). This model remained significant and continued to explain a total variance of 39 percent, *F* [4, 305] = 49.32,* p* < 0.001. The previous PA still had the higher semipartial correlation value (*sr* = 0.29, *p* < 0.001) compared to motivation (*sr* = 0.19, *p* < 0.001), EQ-5D-3L (*sr* = 0.18, *p* < 0.001) and age (*sr* = -0.13, *p* = 0.004).

**Table 3:** Regression result for physical activity with only significant predictors

Variable	B	95% CI for B		SE B	β	R^2^	Δ R^2^
		LL	UL				
Constant	-55.94***	-77.50	-33.00	11.44		.39	.39***
Previous physical activity	10.27***	6.78	13.01	1.72	.33		
EQ-5D-3L	53.26***	26.68	76.73	12.64	.20		
Motivation	5.65***	3.61	7.94	1.10	.22		
Age	-2.73**	-4.42	-1.02	.85	-.14		

*N* = 310. EQ-5D-3L health status. Confidence interval and standard error are based on 1000 bootstrap samples

*CI c*onfidence interval*, LL *lower limit*, UL *upper limit

* *p* <.05. ** *p* < .01. *** *p* < .001

## The qualitative results

We used content analysis to explore the second research question: What do older adults perceive as influencing their levels of PA? The analysis confirmed that all the COM-B subcomponents—physical capability, psychological capability, physical opportunity, social opportunity, reflective motivation, and automatic motivation—were relevant to older adults' PA levels based on the entire sample’s quotes (see Table 4).

**Table 4:** Summary of what older adults’ perceived to influence their physical activity

Category	Subcategory	Example quote
Physical capability	—	*“I have got … these heart problems, I have also gotten this rheumatic muscle disease. It has … automatically made me do less … /// … of these intensive activities … I think so.”* (Participant 1) *“But things that I would like to do, like go to the gym for example, I would like to do that but I have so bad balance so … I cannot do it and at home I have it so nicely arranged with points of support where I can hold on to … and when I walk from the living room through the hall to the kitchen, I have a couple of benches and cupboards, like this one, that I can hold on to …”* (Participant 9)
Psychological capability	Attention	*“… you are not the same person when it comes to attention and other things in the traffic, you are not, it’s a mere fact.”* (Participant 1) *“Sometimes I exercise in the water. Yes, the last time I did it I almost not survived since my sugar level was over 50 … so I exhorted myself in the pool … so I almost did not make it out of it.”* (Participant 5)
	Knowledge	*“There are [activities] in the city center, but then you have to drive and park or ride the bus, and I do not know how to take the bus either. It’s been so many years since I rode a bus, I don’t know how you do it.”* (Participant 5) *“… it’s fashionable to be physically active, I have become more aware of how important it is to move your body.”* (Participant 4)
	Acceptance	*“The idea of aging and not being able to get down the stairs and everything concerning that … I don’t* *agonize over it. I rather look at what is good.”* (Participant 9) *“I am grateful that my body functions. My prime time has passed and now I’ve entered a new era and you do the best you can with it.”* (Participant 3)
Physical opportunity	—	*“… I wouldn’t move a millimeter if I did not have that [the exercise device].”* (Participant 7) *“I couldn’t go on a bus trip yesterday, for example, because I can’t get on a bus with stairs, but I am able to take the city bus because I can get on at certain stops … I have checked the curb’s position in relation to the bus when it lowers its side so I know where I am able to get on and off …”* (Participant 9)
Social opportunity	—	*“We almost never walk if it rains, except for on Thursdays when the group activity is, then we always walk, every time. I think it’s peer pressure that does it. /// I think everyone thinks it’s very nice that we have this activity and that it happens just one day a week. I think that’s just the right amount of activity; otherwise, if you meet people every day you may get bored with them. So, I think it is very perfect actually*.” (Participant 10) *“[My partner] sometimes says … ‘Well, today you have to go out and walk by yourself, because today I don’t have the energy for it. /// Then sometimes you get a bit of guilty conscience, I have to admit, ‘I will do this or that and you can’t, so it’s a balancing act when deciding what activities to engage in.”* (Participant 1)
Automatic motivation	Impulses and inhibition	*“Sometimes I don’t feel like leaving the home to go exercising. Then, of course, I can let it be and I might then do a little bit more at home. But, no, it’s automatic – you get up, eat and get dressed and go over there [the gym]. It has become a habit*.” (Participant 4) *“I’m that kind of person who gets terribly intense when I start something, I don’t give up. I stop when I almost collapse, maybe that’s an exaggeration, but I’m very persistent when I get started.”* (Participant 10)
	Emotions	*“It’s a sorrow to notice that you can’t … first of all, I’ve stopped baking because I’m unable … and now I don’t meet people either …”* (Participant 6) *“It’s practical [to exercise with Sofia on the TV] because it’s at home, but at the same time it’s – Yes, it’s practical because it is at home but at the same time it’s not practical if I say so, in fact, it’s rather boring since you get stuck at home.”* (Participant 10)
	Motives	*“I would rather sit inside and sew than [exercise], like… I’ve never gotten a taste for physical activity, so that it, I do little of it now.”* (Participant 6) *“I want to do it, but the question is whether I can manage to do it.”* (Participant 7)
Reflective motivation	Beliefs	*“No, it doesn’t concern me so much, I just enjoy being able to get out and about to do the activity. That’s what’s important, not how far or how fast I go. The most important thing is that the activity is completed.”* (Participant 2) *“Well, there is a lot of thing you can do, but … no I don’t think that I will start doing those activities that increase the general condition. I will not start jogging or do any other thing like that at this age.”* (Participant 1)
	Goals and plans	*“Yes, but [without movements] I will get stuck and become sedentary, and I already pay 2500 SEK a month for home care and I don’t want to do that. I’m a poor pensioner.”* (*Participant 9)* *“It feels quite hopeless now that I’m not well, but … I know one thing … that is … if I only get better from this … then I’ll start with some activities … a walk, not playing tennis or anything, but maybe swimming at the bathhouse or something … that would be fun.”* (Participant 6)

— = no subcategory identified

### Physical capability

Physical capability concerns how a long life may take a toll on a person’s body and cause mobility issues and focused on *strength and stamina*. The participants described different illnesses and ailments, restrictions in mobility, and a general decreased fitness as reasons for reduced strength and stamina. Common ailments were stiffness, tiredness, and pain, frequently leading to avoidance of actions like running or even walking. Restrictions in mobility caused a range of issues, from being unable to stand up by oneself to not being able to walk at all or only walking with a walker: “/…/ Right now, I do nothing because my back hurts so bad, it's not possible … you see … I can barely get out of the kitchen*"* (Participant 6). In a seemingly downward spiral, decreased general fitness was related to reduced cardio-fitness, strength, and balance. A decreased strength and increased stiffness made lifting items difficult, and stiffness combined with poor balance prevented most physical activities such as biking, dancing, or going to the gym.

### Psychological capability

Psychological capability describes a person’s mental functioning and understanding; three subcategories were identified: *attention*, *knowledge*, and *acceptance*. The first concerns decreased attention in traffic or not noticing body signals (such as low blood sugar levels). The second, mainly referred to a lack of knowledge, not knowing how to get to an activity (for example, being unfamiliar with the bus system), or not knowing what appropriate or available activities would be for their health conditions.

But I want to do something: move my body. In every newspaper and on TV, they say that you should move, but how and where? Who should I turn to? Because I can’t go to a gym since I can't stand up right there. (Participant 7)

The third subcategory, acceptance, was a permissive approach that some participants adopted when faced with lost abilities. This strategy seemed to boost their well-being.

### Physical opportunities

Physical opportunities concern the external living conditions related to *time and inanimate aspects* of an environmental system. Aspects that benefited PA levels included time, financial means, activities to choose between, and access to music, equipment, nature, and facilities. The COVID-19 pandemic, unpleasant weather conditions, and physical surroundings sometimes prevented mobility. For example, the stairs into busses hindered rides or the pavement levels prevented getting on and off a bus everywhere. Dark and slippery roads, as well as long distances, were additional barriers: “/…/ When I walk, it’s to go and play bingo, but then I have to stop several times, and I only wish there were more benches along the pathway for me to sit down for a little while …” (Participant 5).

### Social opportunities

Social opportunities relate to people and cultural elements of an environmental system. These concerned *interpersonal influences,* belonging to a community, and the presence of others who encouraged, pushed, or guided the participants facilitated engagement in PA: “I have actually done [some physical activity]. And if I had someone close by who … liked the training, we could motivate each other, and then it would surely be even more [activity]” (Participant 4). Others’ opinions and actions directed older adults’ behavior. When one partner did not engage in PA, the other often reduced or stopped their activities too. Reasons were feelings of guilt or lack of time due to an increased need for them to do the household chores.

### Automatic motivation

Automatic motivation concerns emotions and impulses that energize behavior. Three subcategories were identified: *impulses and inhibitions, emotions*, and *motives*. The first, impulses and inhibition, concerned relatively unreflective internal forces that propel or restrain actions. Habits of regular exercise or walks were automatic behaviors that facilitated PA. At the same time, a struggle to stop exercising before pain or exhaustion prevented future PA. The second subcategory, emotions, concerns positive and negative feelings for PA. Positive feelings like fun, play, and enjoyment facilitated PA, whereas negative emotions like sadness, fear, and guilt limited activity levels. The fear that restricted behavior was mainly about hurting oneself and losing body functions and abilities.

Like I said, I try to be active ... within my limits. I no longer expose myself to anything extreme, although I feel I should. /…/ This stiffness I have, I wish I didn’t, but to a certain degree, you have to accept it as well when you have passed the age of 80, I think. Perhaps not endangering the body too much. (Participant 2). 

The third subcategory, motives, involved wants and needs that elicited PA and facilitated it as far as mobility issues did not pose a hindrance. The desire to do activities other than PA increased older adults’ motives to be physically active.

Everything comes down to one’s will ... if I don’t want something, I don't want it ... you can’t force someone, it’s quite simple when you come to an understanding ... so ... but sometimes I think about the fact that ... I’ll be 72 years old now, I don’t have damn long left on Earth, and the time I have left I actually want to be a little active. I have no desire to use an electric wheelchair or become a vegetable. (Participant 8)

### Reflective motivation

Reflective motivation involves conscious thinking that can ignite behavior, and two subcategories were identified: *beliefs,* and *goals and plans*. Beliefs concerned ideas about PA, age and age-appropriate manners, and the self. If one believed participating in PA was valuable and worthwhile, it facilitated their engagement. Statements of PA were often accompanied by imperative thoughts, such as ‘must’ or ‘should.’ Regarding beliefs about age, those participants who identified themselves as old tended to perceive PA as too late to engage in, which often restricted their range of activities. Ideas about the self as an active or inactive person influenced the level of PA, where the former facilitated and the latter hindered. “I don't move my body because I have no interest in it” (Participant 5). The subcategory, goals and plans, involved cognitive representations of desired outcomes and intentions for PA. PA helped participants achieve health and independence by reducing illnesses and ailments, lowering restrictions in mobility, improving general fitness, or boosting well-being.

Yes. No, [the goal of these activities] it’s to keep the body going to remain able to cope as I age. I mean, the muscles disappear, the older they get. Of course, you want to try to remain strong and live a long life. I think it’s super-important to exercise because you stay healthier. If you exercise, you have more stamina, and you have fun while exercising. So no, I think it is very important. (Participant 4)

Other goals were to save money and experience nature. A more or less conscious objective with the PA concerned structure and meaning of the days. For example, getting outside the home allowed the participants to explore and be stimulated. Intentions to be active facilitate an active lifestyle and committing to oneself or others further prompted PA. “/… /I take care of dogs and sometimes when I go out, the weather is miserable, but since I’ve promised her, I’ll go out anyway … /…/even if it’s not very tempting to go out when it rains” (Participant 2).

## The mixed-method results

The quantitative results were integrated with the qualitative findings to address the third research question: To what extent do the quantitative results on older adults’ PA levels agree and disagree with the qualitative findings on older adults’ PA levels? This revealed both convergence and divergence (see Table 5).

**Table 5:** Integrated results matrix on factors influencing older adults’ PA level

Quantitative results	Qualitative results					
	Physical capability	Psychological capability	Physical opportunity	Social opportunity	Automatic motivation	Reflective motivation
Motivation					X	X
EQ-5D-3L	X^a^					
Age	X^a^					
Previous physical activity						

X = convergence between factors identified in quantitative and qualitative results. EQ-5D-3L = health status

^*a*^ The resemblance between the measures of physical capability, health status, and age indicates a convergence; that is, body-related abilities (i.e., physical capability) matter to older adults´ PA levels

## Discussion

This mixed-method study utilized quantitative and qualitative methods to examine the factors influencing older adults’ PA levels. This allowed a greater insight than would be obtained by either dataset separately, as quantitative methods enable statistical generalizability to a greater degree and qualitative methods can provide deeper insights and a more nuanced view of a phenomenon [27]. The quantitative analysis of standardized questionnaires was used to identify individual and external factors predicting older adults’ PA levels, and the qualitative analysis of semi-structured interviews was used to gain a better understanding of nuances of what older adults perceive as influencing their PA levels. The mixed-method analysis assessed to what extent the quantitative results on older adults’ PA levels agree and disagree with the qualitative findings on older adults’ PA levels. The discussion will follow the two research questions, answered by quantitative and qualitative methods. After that, an integrated discussion will address the third research question.

### Predictors of PA levels in older adults

The findings revealed that the best predictor for older adults’ PA levels was their previous PA engagement, followed by their current motivation, health status, and age, which aligns with prior research [7, 9, 11, 23–25, 37]. Non-significant variables in the regression analysis were capability, opportunity, subjective SES, and depressive symptoms. Capability changed to a non-significant predictor when health status and age were included, indicating that body-related capacity matters for older adults’ PA levels, which Jancey et al. [14] also postulated. This indicates that physical- rather than psychological capability matters for older adults’ PA levels. Our results contradict previous research by not identifying opportunity as a significant predictor [22–25]. This may be due to multicollinearity and other factors that matter more to older adults’ PA levels. Additionally, the availability of walking groups in their residential areas may have impacted participants’ perceptions of opportunities, influencing the results. Another possibility is range restriction in the participants’ questionnaire answers in this study or that other research studies have used different items to measure the COM-B variables.

Despite previous negative associations between subjective SES with physical inactivity [16] and mobility issues [17], subjective SES was not a significant predictor in our study. The reason could be the homogeneity in the sample, with all participants living in rented apartments reporting fairly similar subjective SES.

Many older adults suffer from depression and in Sweden approximately 10 percent of individuals aged 65–74 use antidepressants, increasing to nearly 20 percent for those aged 85–94 [38]. The prevalence of depression in our sample (potentially 24.4%) is higher than that of Sweden; however, it is lower than the global equivalent (28.4%) [39]. Depressive symptoms were not a significant predictor of PA levels, although previous research has shown that depressive symptoms may hinder PA engagement [18]. The discrepancy in findings may be due to different study designs and sample sizes. Our quantitative study included 334 participants in a single measurement, whereas Lindwall et al. [18] employed a repeated measurement design with a sample of almost 18,000 participants. In summary, early engagement in PA seems to be a precursor for maintaining an active lifestyle later in life. Still, other factors like the current motivation and age-related health status are also important.

### What do older adults perceive as influencing their PA levels?

All subcomponents of the COM-B model were identified as relevant to help understand what influences PA levels among older adults, which validates the results from previous reviews [22, 25]. As with all models, the COM-B model is a simplified representation of reality, and the subjectivity in data interpretation might account for differences in findings among researchers. Meredith et al. [25], who reviewed qualitative studies on older adults’ PA participation and mapped their findings to the COM-B model, perceived fear as an individual vulnerability related to capability. However, we interpreted it as an emotion related to automatic motivation. Nonetheless, our findings and Meredith et al. [25] suggest that fear of falling or losing physical abilities can limit PA.

It is well known that older adults can face challenges in PA due to reduced strength and stamina, especially if the social and physical environments do not support their health condition. Our results show that all the COM-B model’s subcomponents are relevant and have complex interactions. This has also been acknowledged by Meredith et al. [25], who reported that a greater portion of their findings was related to social and physical opportunities. Other researchers, who did not only include older adults in their samples, have emphasized physical opportunity while downplaying physical capability and social opportunity [22, 24]. This suggests that social environment and physical capabilities become more significant later in life and implicates the importance of analyzing age groups separately, as differences can become camouflaged when combined. In summary, the subcomponents of the COM-B model help explain older adults’ engagement or withstanding of PA. The importance of age-disaggregated analysis is also revealed when comparing our results on older adults to those of other researchers that include samples with not only older adults.

### Integrative discussion

The quantitative and qualitative results were both convergent and divergent (see Table 5). In the quantitative results, opportunity was not a significant predictor, but it was identified as an influential category in the qualitative findings. This discrepancy may be due to how opportunity was measured in the quantitative analysis. The qualitative analysis revealed additional nuances of opportunity that were not assessed in the survey. For example, the qualitative analysis suggested that PA levels are influenced by the existence of PA facilities as well as the physical surroundings that govern how a person can access the facility. Therefore, participants may have responded in the survey that facilities for exercising existed without considering if the physical surroundings allowed them to access them. Similarly, the quantitative survey did not include any item on how the ability of a partner to engage in PA affected the participant's opportunity to be active, which was reported as influential in the qualitative analysis. Nonetheless, this divergence calls for more research regarding the role of opportunity for older adults’ PA levels. Regarding capability, the quantitative analysis only reveals physical capability as core to older adults’ PA levels, while both physical- and psychological capability are identified in the qualitative analysis. Motivation was recognized as central in both analyses. The qualitative findings nuanced the quantitative results by indicating both reflective and automatic motivation as relevant. This life-span perspective concerns a distinction between the two analyses, as previous PA was a statistically significant predictor in the quantitative results but did not surface as a category in the qualitative findings. During the interviews, some participants talked about being active in the past, but they did not associate it with their current PA levels. This divergence between the datasets may be related to the qualitative data collection’s focus on individual experiences, while the quantitative approach emphasizes patterns in large groups.

Comparing the quantitative and qualitative data can improve our understanding of what influences PA in older adults. Our findings show that, as people age, their behavior and cognition change, as does their motivation to engage in PA. Pleasurable, meaningful, and social activities reinforce older adults’ PA positively. However, as shown in this study, aging deteriorates a person’s body and can restrict their current PA levels. In these situations, the surrounding opportunities and the individuals’ knowledge of safely engaging in PA matters. Also, people’s previous experience of PA influences their present behavior. In other words, our study indicates that many factors influence older adults’ PA levels in a complex manner. The COM-B model and its subcomponents seem like a relevant model for understanding older adults’ PA levels. In summary, these findings suggest that applying a life-span perspective and considering the COM-B model's subcomponents can help explain why older adults engage in PA or not.

### Implications and practical significance

Stakeholders may promote healthy aging and contribute to the 2030 Agenda’s sustainable developmental goal of health and well-being [8] by utilizing knowledge of factors influencing PA levels in older adults. It is important to recognize that aging can look very different from one person to another, and that this heterogeneity tends to increase with age, peaking at approximately 70 years for various health characteristics [40]. Our findings reveal both hetero- and homogeneity among the participants. For example, in the qualitative analysis, they all reported reduced strength and stamina, but the reasons varied from biological to behavioral. The individual differences must be considered, but common features allow for PA promotions for healthy aging.

Firstly, this result implies the importance of prioritizing PA at early life stages, as this positively affects PA levels in older adults. However, this alone will not suffice to increase PA levels in the aging population, as their past cannot be changed. Hence, interventions to target PA in older adults are necessary.

Secondly, interventions to increase PA among older adults should review all subcomponents of the COM-B model. This knowledge is valuable since the COM-B model is the hub of The Behavior Change Wheel (BCW) and can be used to develop and evaluate interventions [19]. According to the BCW, interventions should be developed systematically, and the first stage is understanding the behavior through the COM-B lens [19]. This can be considered to have been achieved by the present study and the previous study of Meredith et al. [25]. In the second stage of intervention development; stakeholders can consult the BCW [19] while using the results from this study to create interventions that are specific to their settings to promote PA among older adults. For example, our research indicates that in addition to providing PA facilities, successful PA intervention may also require attention to the physical surroundings or the person’s knowledge of how to use the facility.

### Limitations, strengths, and future research

The mixed-method design is a strength, as two data sets allow a thorough assessment of what influences older adults’ PA levels. For example, the quantitative analysis identified previous PA as an important influencer to older adults’ PA, which is not emphasized in our qualitative analysis nor by Meredith et al. [25]. Our results regarding the COM-B model validate Meredith et al. [25] findings. It can, therefore, be concluded that the COM-B model is useful for understanding older adults’ PA. This is valuable information since the COM-B related results can conveniently be transformed into interventions due to its connection with the framework of (BCW) [19]. Another strength of this study is that our analysis sheds light on the importance of age-disaggregated research when our findings, related to an older population, are compared to prior studies [22, 24] that not only include older adults. Future research should preferably age disaggregate their analysis by the recommended five-year age brackets [41].

A limitation concerns the findings' generalizability or transferability since the drop-out rate was rather large in the quantitative sample from which the qualitative sample was recruited. Additionally, not everyone invited to the qualitative study consented to participate, meaning that those who chose to participate in this mixed-method study may differ from non-participants. However, the sample size in both datasets was relatively sizable, which boosts the statistical power of the quantitative analysis and nuances in the verbal statements in the qualitative analysis. The living conditions of the older adults, included in our study, may differ slightly from other samples used in prior research, challenging result comparisons. Future studies are recommended to include a more diverse population of older adults. Additionally, our sampling resulted in different mode ages in the two samples, which is a limitation as it may mirror different realities among older adults, thereby potentially influencing the result integration. Another weakness within the quantitative dataset concerns the precision of measurement, multicollinearity, and range restriction for the included variables in the regression analysis. A potential limitation of the qualitative study was the lack of member checks. However, to compensate for this, the participants were invited to contact the researchers with adjustments or additional comments on their interviews. To further ensure credibility and trustworthiness, all interviews were conducted within two months. Additionally, multiple interviewers and coders from different disciplines (i.e., investigator triangulation) helped minimize research bias, enhancing the qualitative analysis. Some talkative participants occasionally strayed off-topic, which may have influenced the data collection and the qualitative findings.

The qualitative analysis indicated the importance of PA as a pleasurable activity and previous research has associated subjective well-being [42] and morale [43] with older adults’ PA. However, the quantitative analysis did not include these emotional aspects as variables. We suggest that future studies review and clarify the value of positive and negative emotions for older adults’ PA levels. Additionally, longitudinal experimental study designs are needed to clarify the role of physical- and social opportunities for older adults’ PA.

## Conclusion

To our knowledge, this study is the first to use a convergent mixed-method design to examine factors influencing PA levels in older adults aged 65 and above who rent apartments from the municipality with access to community hosts, providing a more comprehensive understanding of the topic. It seems that many factors influence older adults’ PA levels in a complex manner, with the integrated result showing convergence regarding motivation and physical capability but divergence in psychological capability, opportunity, and previous PA engagement. The findings also indicate relevance to the COM-B model as a framework for understanding older adults’ PA levels. Overall, we suggest that it is important to consider all the COM-B model’s subcomponents when designing a PA program for older adults and to apply a life-span perspective, as previous PA engagement seems to influence the current level of PA in older adults. However, it is also central to consider their current motivation, capability, and opportunities to understand what influences their PA levels. More research is needed to clarify the role of emotions and opportunities for older adults’ PA levels since the findings are inconsistent. Furthermore, the value of age-disaggregated data is revealed when our findings from samples of only older adults are compared to previous research that does not only include older people.

From a public health perspective, prioritizing PA early in life appears important, as this can positively impact older adults’ PA engagement. Based on our findings, we would make the following recommendations for promoting PA among older adults. Since the findings can be related to the intervention framework of BCW, stakeholders are encouraged to use these results while also seeking further guidance from the BCW to design interventions to improve PA levels and promote healthier aging among older adults. For instance, our findings suggest that it is important to consider the targeted population's physical abilities and offer appropriate options for their health condition when designing an intervention. Also, to alleviate the fear of injury that can hinder motivation for PA, it is central to address older adults’ concerns and provide them with the necessary knowledge to engage in PA safely. The findings also indicate the importance of PA to be fun, playful, and meaningful. This knowledge can be used to frame and present PA options to participants to motivate PA engagement. Challenging age stereotypes and emphasizing that it is never too late to start exercising appears also important. Additionally, to ensure the success of PA interventions, it is also important to consider the physical surroundings and social settings at macro and meso levels. For example, long walking distances with no resting spots or a partner's physical ability may prevent PA engagement. Addressing such issues can help individuals to partake in activities without limitations.

## Data Availability

The datasets used and analyzed during the current study are available from the corresponding author upon reasonable request.

## Abbreviations

PA: Physical activity
Subjective SES: Subjective socioeconomic status
BCW: The Behavior Change Wheel

## References

[1] Eckstrom E, Neukam S, Kalin L, Wright J. Physical activity and healthy aging. Clin Geriatr Med. 2020;36(4):671–83. 33010902 10.1016/j.cger.2020.06.009

[2] Moreno-Agostino D, Daskalopoulou C, Wu Y-T, Koukounari A, Haro JM, Tyrovolas S, et al. The impact of physical activity on healthy ageing trajectories: Evidence from eight cohort studies. Int J Behav Nutr Phys Act. 2020;17(1):92. 10.1186/s12966-020-00995-8 PMC7364650 32677960

[3] WHO. WHO guidelines on physical activity and sedentary behaviour 2020 [Available from: https://iris.who.int/bitstream/handle/10665/336656/9789240015128-eng.pdf?sequence=1andisAllowed=y. 33369898

[4] WHO. Global recommendations on physical activity for health 2010 [Available from: https://iris.who.int/bitstream/handle/10665/44399/9789241599979_eng.pdf?sequence=1&isAllowed=y. 26180873

[5] Wang H, King B. Understanding community-dwelling Chinese older adults’ engagement in physical activity: a grounded theory study. Gerontologist. 2022;62(3):342–51. 34023876 10.1093/geront/gnab069

[6] Public Health Agency of Sweden. Riktlinjer för fysisk aktivitet och stillasittande: Kunskapsstöd för främjande av fysisk aktivitet och minskat stillasittande 2021 [Available from: https://www.folkhalsomyndigheten.se/contentassets/106a679e1f6047eca88262bfdcbeb145/riktlinjer-fysisk-aktivitet-stillasittande.pdf.

[7] Sun F, Norman IJ, While AE. Physical activity in older people: a systematic review. BMC Public Health. 2013;13(1):449. 10.1186/1471-2458-13-449 PMC3651278 23648225

[8] UN General Assembly. Transforming our world: The 2030 agenda for sustainable development 2015 [Available from: https://sustainabledevelopment.un.org/post2015/transformingourworld/publication.

[9] Castillo JMD, Navarro JEJ-B, Sanz JLG, Rodríguez MM, Izquierdo AC, Pinés DDH. Being physically active in old age: Relationships with being active earlier in life, social status and agents of socialization. Ageing and Society. 2010;30(7):1097–113.

[10] Barbaccia V, Bravi L, Murmura F, Savelli E, Viganò E. Mature and older adults’ perception of active ageing and the need for supporting services: Insights from a qualitative study. Int J Environ Res Public Health. 2022;19(13):7660. 10.3390/ijerph19137660 PMC9265376 35805320

[11] Sansano-Nadal O, Giné-Garriga M, Rodríguez-Roca B, Guerra-Balic M, Ferri K, Wilson JJ, et al. Association of self-reported and device-measured sedentary behaviour and physical activity with health-related quality of life among european older adults. Int J Environ Res Public Health. 2021;18(24):13252. 10.3390/ijerph182413252 PMC8706726 34948861

[12] Olivares PR, Gusi N, Prieto J, Hernandez-Mocholi MA. Fitness and health-related quality of life dimensions in community-dwelling middle aged and older adults. Health Qual Life Outcomes. 2011;9:117. 10.1186/1477-7525-9-117 PMC3286398 22192520

[13] Huffman MK, Amireault S. What keeps them going, and what gets them back? Older adults’ beliefs about physical activity maintenance. Gerontologist. 2021;61(3):392–402. 32622348 10.1093/geront/gnaa087

[14] Jancey JM, Clarke A, Howat P, Maycock B, Lee AH. Perceptions of physical activity by older adults: A qualitative study. Health Educ J. 2009;68(3):196–206.

[15] Janssen SL, Stube JE. Older adults’ perceptions of physical activity: a qualitative study. Occup Ther Int. 2014;21(2):53–62. 24302685 10.1002/oti.1361

[16] Shankar A, McMunn A, Steptoe A. Health-related behaviors in older adults relationships with socioeconomic status. Am J Prev Med. 2010;38(1):39–46. 20117555 10.1016/j.amepre.2009.08.026

[17] Hu P, Adler NE, Goldman N, Weinstein M, Seeman TE. Relationship between subjective social status and measures of health in older Taiwanese persons. J Am Geriatr Soc. 2005;53(3):483–8. 15743294 10.1111/j.1532-5415.2005.53169.x

[18] Lindwall M, Larsman P, Hagger MS. The reciprocal relationship between physical activity and depression in older European adults: a prospective cross-lagged panel design using SHARE data. Health Psychol. 2011;30(4):453–62. 21480713 10.1037/a0023268

[19] Michie S, Atkins L, West R. The behaviour change wheel: A guide to designing interventions. Sutton: Silverback Publishing; 2014.

[20] Campbell M, Fitzpatrick R, Haines A, Kinmonth AL, Sandercock P, Spiegelhalter D, et al. Framework for design and evaluation of complex interventions to improve health. BMJ. 2000;321(7262):694–6. 10987780 10.1136/bmj.321.7262.694 PMC1118564

[21] Michie S, van Stralen MM, West R. The behaviour change wheel: A new method for characterising and designing behaviour change interventions. Implementation Science. 2011;6(1):42. 10.1186/1748-5908-6-42 PMC3096582 21513547

[22] Knight RL, McNarry MA, Sheeran L, Runacres AW, Thatcher R, Shelley J, et al. Moving forward: Understanding correlates of physical activity and sedentary behaviour during covid-19—an integrative review and socioecological approach. Int J Environ Res Public Health. 2021;18(20):10910. 10.3390/ijerph182010910 PMC8535281 34682653

[23] Roche C, Fisher A, Fancourt D, Burton A. Exploring barriers and facilitators to physical activity during the COVID-19 pandemic: A qualitative study. Int J Environ Res Public Health. 2022;19(15):9169. 10.3390/ijerph19159169 PMC9367830 35954538

[24] Pelletier C, Cornish K, Amyot T, Pousette A, Fox G, Snadden D, et al. Physical activity promotion in rural health care settings: a rapid realist review. Prevent Med Rep. 2022;29:29. 10.1016/j.pmedr.2022.101905 PMC9307466 35879935

[25] Meredith SJ, Cox NJ, Ibrahim K, Higson J, McNiff J, Mitchell S, et al. Factors that influence older adults' participation in physical activity: A systematic review of qualitative studies. Age and Ageing. 2023;52(8). 10.1093/ageing/afad145 PMC10438214 37595070

[26] Creswell JW, Plano Clark VL. Designing and conducting mixed methods research. 3 ed. Thousand Oaks, CA: SAGE; 2017.

[27] Cohen L, Manion L, Morrison K. Research methods in education. 6: ed. Routledge; 2007.

[28] Statistics Sweden. Boende i Sverige 2023 [Available from: https://www.scb.se/hitta-statistik/sverige-i-siffror/manniskorna-i-sverige/boende-i-sverige/.

[29] Godin G. The godin-shephard leisure-time physical activity questionnaire. Health Fitness J Can. 2011;4(1):18–22.

[30] Gottfries GG, Noltorp S, Nørgaard N. Experience with a Swedish version of the geriatric depression scale in primary care centres. Int J Geriatr Psychiatry. 1997;12(10):1029–34. 9395935 10.1002/(sici)1099-1166(199710)12:10<1029::aid-gps683>3.0.co;2-d

[31] Bäccman C, Bergkvist L, Wästlund E. Personalized coaching via texting for behavior change to understand a healthy lifestyle intervention in a naturalistic setting: Mixed methods study. JMIR Format Res. 2023;7:e47312. 10.2196/47312 PMC10687691 37966893

[32] EuroQol Research Foundation. EQ-5D-3L User Guide: Basic information on how to use the EQ-5D-3L instrument. Version 6.0.; 2018.

[33] Burström K, Sun S, Gerdtham U-G, Henriksson M, Johannesson M, Levin L-Å, et al. Swedish experience-based value sets for EQ-5D health states. Qual Life Res. 2014;23(2):431–42. 23975375 10.1007/s11136-013-0496-4 PMC3967073

[34] Adler NE, Epel ES, Castellazzo G, Ickovics JR. Relationship of subjective and objective social status with psychological and physiological functioning: reliminary data in healthy. White Women Health Psychol. 2000;19(6):586–92. 11129362 10.1037//0278-6133.19.6.586

[35] Hsieh H-F, Shannon SE. Three approaches to qualitative content analysis. Qual Health Res. 2005;15(9):1277–88. 16204405 10.1177/1049732305276687

[36] Graneheim UH, Lundman B. Qualitative content analysis in nursing research: concepts, procedures and measures to achieve trustworthiness. Nurse Educ Today. 2004;24(2):105–12. 14769454 10.1016/j.nedt.2003.10.001

[37] Zhang J, Bloom I, Dennison EM, Ward KA, Robinson SM, Barker M, et al. Understanding influences on physical activity participation by older adults: A qualitative study of community-dwelling older adults from the Hertfordshire Cohort Study, UK. PLoS ONE. 2022;17(1):e0263050. 10.1371/journal.pone.0263050 PMC8789143 35077522

[38] The National Board of Health and Welfare. Kartläggning och analys av föreskrivning av antidepressiva läkemedel till personer 65 år och äldre 2023 [Available from: https://www.socialstyrelsen.se/globalassets/sharepoint-dokument/artikelkatalog/ovrigt/2023-11-8828.pdf.

[39] Hu T, Zhao X, Wu M, Li Z, Luo L, Yang C, et al. Prevalence of depression in older adults: A systematic review and meta-analysis. Psychiatry Res. 2022;311:114511. 10.1016/j.psychres.2022.114511 35316691

[40] Nguyen QD, Moodie EM, Forget M, Desmarais P, Keezer MR, Wolfson C. Health heterogeneity in older adults: exploration in the Canadian longitudinal study on aging. J Am Geriatr Soc. 2021;69(3):678–87. 33155270 10.1111/jgs.16919

[41] WHO. UN decade of healthy ageing: Plan of action (2021–2030) 2020 [Available from: https://cdn.who.int/media/docs/default-source/decade-of-healthy-ageing/decade-proposal-final-apr2020-en.pdf.

[42] Chen S, Calderón-Larrañaga A, Saadeh M, Dohrn I-M, Welmer A-K. Correlations of subjective and social well-being with sedentary behavior and physical activity in older adults-A population-Based study. J Gerontol A Biol Sci Med Sci. 2021;76(10):1789–95. 33674835 10.1093/gerona/glab065 PMC8436992

[43] Almevall AD, Wennberg P, Zingmark K, Öhlin J, Söderberg S, Olofsson B, et al. Associations between everyday physical activity and morale in older adults. Geriatr Nurs. 2022;48:37–42. 36099778 10.1016/j.gerinurse.2022.08.007

